# Sequential conditioning in hematopoietic stem cell transplantation in elderly high-risk myeloid blood cancer patients: long-term survival, disease control, and immune recovery across donor types in a matched-pair analysis

**DOI:** 10.3389/fimmu.2026.1803250

**Published:** 2026-08-26

**Authors:** Sarah Haebe, Elena Stauffer, Heidrun Drolle, Dusan Prevalsek, Moritz Schmidt, Quentin Fichaux, Michael Weigand, Alessia Fraccaroli, Johanna Tischer

**Affiliations:** 1 Department of Medicine III, Ludwig Maximilian University Hospital Munich, Munich, Germany; 2 Institute of Laboratory Medicine, Ludwig Maximilian University Hospital, Munich, Germany

**Keywords:** AML, GvHD, immune reconstitution, MDS, NRM, sequential conditioning, long-term survival, allogeneic transplantation

## Abstract

**Background:**

Sequential conditioning allogeneic hematopoietic stem cell transplantation (allo-HSCT) regimens might offer improved disease control in high-risk myeloid patients. But concerns remain regarding toxicity, infection risk and delayed immune reconstitution (IR), particularly in older and HLA-haploidentical transplant recipients. To address these concerns, we comprehensively compared safety, feasibility and clinical outcome across HLA-matched related (MRD), matched unrelated (MUD) and haploidentical donor (Haplo) HSCT and longitudinally characterize IR patterns and their associations with post-transplant outcomes.

**Methods:**

We conducted a retrospective matched-pair analysis comparing MRD-, matched MUD- and Haplo-HSCT in elderly patients matched for (1) disease activity: p = 1.0; (2) disease status: p = 1.0; (3) modified disease risk index (DRI): p = 0.9; (4) hematopoietic cell transplantation comorbidity index (HCT-CI): p = 0.92; and (5) age: p = 0.95. Outcomes included disease-free (DFS) and overall survival (OS), relapse, non-relapse mortality (NRM), graft-versus-host disease (GvHD), toxicity, infections, and donor-specific IR dynamics and their impact on clinical outcomes.

**Results:**

With a median follow-up of more than 9 years, no significant differences were observed in long-term disease control and survival among the three groups (5-y DFS/OS: MRD = 41%/41%, MUD = 56%/63%, Haplo = 58%/58%; p = 0.52/0.55). Cumulative incidences (CI) of 5-y-NRM and 1-y moderate and severe chronic GvHD (cGvHD) rates were comparable among the three groups (NRM/cGvHD: MRD = 19%/13%, MUD = 29%/6%, Haplo = 18%/19%; p = 0.3/0.57). Overall immune cell recovery after one year was comparable among groups, though early recovery of CD3+ T and NK cells was delayed after Haplo-HSCT. Subgroup analysis revealed that higher early CD4+ T and lower B cell counts were associated with increased CI of acute GvHD III-IV° (p = 0.04/0.03). Additionally, NK recovery at one year was associated with improved DFS and OS as well as lower relapse incidence.

**Conclusions:**

Sequential therapy is safe and feasible in elderly patients with high-risk or active disease across all three transplant platforms. The observed associations between IR and clinical outcomes highlight the relevance of immune monitoring for anticipating post-transplant complications and guiding individualized prophylactic and therapeutic strategies. Prospective studies are needed to further investigate these findings and define the underlying mechanisms.

## Introduction

Sequential regimens combining cytoreductive chemotherapy followed by reduced-intensity conditioning (RIC) have shown promising results in patients with high-risk myeloid disease, particularly acute myeloid leukemia (AML) and myelodysplastic syndrome (MDS) (1, 2). This sequential approach offers effective leukemia control and reduced relapse rates in both HLA-matched and HLA-haploidentical (Haplo) hematopoietic stem cell transplantation (HSCT) settings (1, 3–6). However, despite encouraging clinical outcomes, concerns remain regarding toxicity and infections with sequential conditioning approaches especially in older patients. Similarly, prior studies suggest an elevated infection risk following Haplo-HSCT (7, 8).

A crucial factor in determining clinical outcomes, and in particular infection rates, is immune reconstitution (IR) after allogeneic HSCT (allo-HSCT). IR is influenced by various factors including conditioning regimes, sex, age and graft-versus-host disease (GvHD) prophylaxis. Moreover, the type of donor is thought to significantly influence the pace and quality of IR. In HLA-matched HSCT, rapid IR offers protection against both infections and relapse (9). In contrast, Haplo-HSCT is generally associated with delayed IR compared to HLA-matched related donor (MRD) transplants (10–12). However, comparative data on IR across different transplant modalities and donor types – and in particular the influence of sequential conditioning - remain relatively sparse, largely due to limited granularity of IR data in transplant registries (13). Thus, whether sequential conditioning in the Haplo-HSCT setting compromises IR and increases the risk of infection remains unclear. This question is especially relevant in older patients, where treatment decisions must balance optimal disease control with potential infectious complications.

To address this question, we conducted a retrospective matched-pair analysis comparing sequential conditioning MRD-, matched unrelated donor (MUD)-, and Haplo-HSCT in elderly patients with high-risk myeloid diseases. For better comparison across the three groups, our analysis accounted for confounding factors such as patient age, disease status and activity as well as co-morbidity indices. We evaluated long-term survival and disease-free outcomes as well as GvHD, toxicity, and infection profiles, along with a detailed comparison of cellular IR across the different donor types and its potential impact on clinical outcomes.

## Methods and materials

### Study design

Eligible were older patients (≥ 50 years) with high-risk AML defined by either high-risk cytogenetics, relapsed/refractory AML or untreated secondary/therapy-related AML or high-risk MDS defined by adverse cytogenetics, who underwent their first sequential RIC transplantation at our center between January 2009 and June 2017 using an MRD, MUD (fully allele-match at HLA-A, HLA-B, HLA-C, HLA-DRB1 and HLA-DQB1 loci) or related, haploidentical donor (Haplo; mismatched for ≥ 2 HLA loci mentioned beforehand). Older patients were defined as ≥50 years due to their increased risk of toxicity with sequential conditioning regimens (14). Pre-defined matching criteria for this study included (i) disease activity (blast yes/no), (ii) disease status at start of sequential conditioning (relapse, refractory, high-risk), (iii) modified disease risk index (DRI (15), (iv) hematopoietic cell transplantation specific comorbidity index (HCT-CI), missing closing parentheses (16), and (v) age (+/-5years).

The study was conducted in accordance with German legislation and the revised Helsinki declaration, and was evaluated and approved by the local ethics committee of the Ludwig-Maximilians University of Munich (#19-368). Written informed consent for participation in this retrospective study was obtained from all patients prior to transplantation.

### Conditioning and post-transplant management

Sequential conditioning consisted of either FLAMSA (fludarabine 30 mg/m^2^, cytarabine 1–2 g/m^2^, amsacrine 100 mg/m^2^ IV over 4 days) or clofarabine (30 mg/m^2^ IV over 5 days) prior to RIC. HLA-typing, donor selection as well as supportive microbial prophylaxis were performed as previously described (1). *In vivo* T cell depletion with anti-thymocyte globulin (ATG) was used in the HLA-matched setting (3x10mg/kg in MRD-HSCT, 3x20 mg/kg in MUD-HSCT). Post-transplantation cyclophosphamide (PTCY) was applied in the haploidentical setting (17). Post-grafting immunosuppression consisted uniformly of a calcineurin inhibitor (CNI) and mycophenolate mofetil (MMF). In the HLA-matched setting, ciclosporin A was started on day –1 and MMF on day 0, with planned discontinuation at day +100. In the haploidentical setting, tacrolimus and MMF were both initiated on day +5, with scheduled discontinuation starting at day +150. Post-grafting G-CSF was administered only in the Haplo setting. Letermovir prophylaxis was not used routinely because it had not yet received regulatory approval in Germany at the time. Donor lymphocyte infusions (DLIs) were given in the absence of acute GvHD (aGvHD), either prophylactically or preemptively, for relapse prevention. Following the strategy described by Schmid et al. (18), prophylactic DLIs were given four weeks after cessation of immunosuppression. The timing of immunosuppression withdrawal varied according to the donor platform used.

### Definitions

Genetic aberrations were classified according to the European LeukemiaNet (ELN) (19) or per revised international prognostic scoring system (IPSS-R) (20). Complete remission (CR) prior to allo-HSCT was defined according to standard morphologic criteria. Refractory was defined as disease persistence after induction chemotherapy regimen. Response rate was assessed at day +30 post HSCT through bone marrow (BM) aspiration and chimerism analysis. aGvHD was classified using the established criteria (21, 22); chronic GvHD (cGvHD) was graded following the National Institutes of Health consensus criteria (23, 24). GvHD-free/relapse-free survival (GRFS) and cGvHD-free/relapse-free survival (CRFS) were defined as previously described (25) and considered as secondary endpoints. Non-hematologic toxicities were scored according to National Cancer Institute Common Terminology Criteria for Adverse Events (NCI-CTCAE; version 5.0) from start of sequential therapy until day +30 post allo-HSCT.

### Immune reconstitution

To characterize immune reconstitution, fresh peripheral blood samples collected at predefined time points prior to and after allo-HSCT (+30, +100, +180 and +360 days) were assessed using FACS Canto II flow cytometry (BD). The following fluorochrome-conjugated monoclonal antibodies were used for the identification of immune cell subsets: CD45 (clone 2D1, V500-C, BD), CD3 (clone SK7, APC-H7, BD), CD4 (clone SK3, PerCP-Cy5.5, BD), CD8 (clone SK1, FITC, BD), CD16 (clone B73.1, PE, BD), CD56 (clone MY31, PE, BD), and CD19 (clone SJ25C1, V450, BD). Leukocyte populations were initially gated based on CD45 expression (CD45+). Within this population, T cells were identified as CD3+ cells and further subdivided into CD4+ helper T cells (CD3+/CD4+) and CD8+ cytotoxic T cells (CD3+/CD8+). Natural killer (NK) cells were defined as CD3-/CD16+/CD56+ cells, and B cells were identified as CD19+ cells. Absolute counts and relative frequencies of these immune cell subsets were quantified at each time point to assess the kinetics of immune reconstitution following allo-HSCT.

### Statistical analysis

Survival probabilities were estimated using the Kaplan-Meier method. Death from any cause was considered an event; surviving patients were censored at last follow-up. Cumulative incidence curves accounted for competing risks (e.g. aGvHD, cGvHD, non-relapse mortality (NRM), relapse) and were compared using Gray’s test. Patient and transplant characteristics were compared using the x^2^–test or Fisher’s exact test for categorical variables and the Kruskal-Wallis test for continuous variables. Continuous variables were reported as medians with ranges, categorical variables as percentages. Analyses of IR and its association with clinical endpoints were exploratory and hypothesis-generating. Reported p-values are descriptive and were not adjusted for multiple comparisons, and the possibility of chance findings cannot be excluded; cut-offs for IR markers were determined based on medians and/or quartiles. All tests were two-sided; P<0.05 was considered statistically significant. Analyses were performed using R software (http://www.R-project.org).

## Results

### Patient, disease and transplant characteristics

Out of a total of 34 patients ≥ 50 years with high-risk AML/MDS undergoing sequential conditioning Haplo-HSCT, 17 Haplo patients were successfully pair-matched with 17 MRD and 17 MUD recipients, based on the following matching criteria: (i) disease activity: p = 1.0, (ii) disease status: p = 1.0, (iii) modified DRI: p = 0.9, (iv) HCT-CI score: p = 0.92 and (v) age: p = 0.95.

Median age was 61 years (50–70 years) and the distribution of AML and MDS was similar across donor groups (**Table 1**). Most patients (71%) presented with active disease at the start of cytoreductive therapy. The transplant-related differences relevant for IR included the chemotherapeutic regimen used for sequential conditioning (p = 0.01), the use of PTCY-based GvHD prophylaxis (p<0.01) and a higher proportion of bone marrow grafts in the Haplo setting (p<0.01; **Table 2**). In contrast, MRD/MUD recipients uniformly received FLAMSA-RIC as preparative regimen, PBSCs as graft source, and ATG-based prophylaxis. Other baseline variables were well balanced across the three groups (**Tables 1**, **2**).

**Table 1:** Patient characteristics.

	All	MRD	MUD	Haplo	P-value
Patients	51 (100)	17 (100)	17 (100)	17 (100)	
Patient age*					0.95
Median (range)	61 (50-70)	59 (50-68)	61 (53-70)	61 (52-69)	
Patient sex					0.28
Male	23 (45)	9 (53)	9 (53)	5 (29)	
Female	28 (55)	8 (47)	8 (47)	12 (71)	
Diagnosis*					0.90
AML *de novo*	28 (55)	8 (47)	9 (53)	11 (65)	
sAML	8 (16)	4 (24)	3 (18)	1 (6)	
tAML	6 (12)	2 (12)	2 (12)	2 (12)	
MDS	9 (18)	3 (18)	3 (18)	3 (18)	
Disease stage*					1.0
PIF	9 (18)	3 (18)	3 (18)	3 (18)	
relapse	15 (29)	5 (29)	5 (29)	5 (29)	
high-risk cytogenetics	24 (47)	8 (47)	8 (47)	8 (47)	
untreated	3 (6)	1 (6)	1(6)	1 (6)	
Status prior to HSCT*					1.0
Active disease	36 (71)	12 (71)	12 (71)	12 (71)	
CR	15 (29)	5 (29)	5 (29)	5 (29)	
Modified DRI*					0.9
Intermediate risk	13 (25)	4 (24)	4 (24)	5 (29)	
High risk	27 (53)	8 (47)	10 (59)	9 (53)	
Very high risk	11 (22)	5 (29)	3 (18)	3 (18)	
HCT-CI score*					0.92
0-2	32 (63)	11 (65)	10 (59)	11 (65)	
> 2	19 (37)	6 (35)	7 (41)	6 (35)	

*Served as matching criteria.

Values in parentheses represent percentages if not indicated otherwise.

*AML*, acute myeloid leukemia; *DRI*, disease risk index; *Haplo*, haploidentical donor; *HCT-CI score*, hematopoietic cell transplantation-specific comorbidity index; *MDS*, myelodysplastic syndrome; *MRD*, matched related donor; *MUD*, matched unrelated donor; *PIF*, primary induction failure; *sAML*, secondary AML; *tAML*, therapy-related AML.

**Table 2:** Donor and transplant characteristics.

	All	MRD	MUD	Haplo	P-value
Patients	51 (100)	17 (100)	17 (100)	17 (100)	
Donor age					< 0.01
Median (range)	39 (21-69)	57 (49-69)	34 (23-50)	29 (21-66)	
Donor sex					0.35
Male	29 (57)	8 (47)	12 (71)	9 (53)	
Donor sex-match					0.13
Patient male/donor female	9 (18)	6 (35)	1 (6)	2 (12)	
Patient female/donor male	15 (29)	5 (29)	4 (23)	6 (35)	
Match	27 (53)	6 (35)	12 (71)	9 (53)	
No of HLA mismatches					< 0.01
Median (range)	0 (0-7)	0 (0)	0 (0)	5 (5-7)	
Time to allo-HSCT					0.37
Median months	2 (0-8)	2 (0-4)	2 (0-6)	2 (1-8)	
ABO match	28 (55)	12 (71)	4 (24)	12 (71)	< 0.01
CMV match	37 (73)	15 (88)	11 (65)	11 (65)	0.30
Cytoreduction regimen					0.01
FLAMSA	47 (92)	17 (100)	17 (100)	13 (76)	
Clofarabine	4 (8)	0 (0)	0 (0)	4 (24)	
Stem cell source					< 0.01
BM	9 (18)	0 (0)	0 (0)	9 (53)	
PBSC	42 (82)	17 (100)	17 (100)	8 (47)	
Median cell dose (range)					n.a
NCx10^8^/kg BW	-–	-–	-–	2.7 (2-4)	
CD34x10^6^/kg BW	7.7(4-17)	9 (5-14)	8 (4-15)	5.6 (4-17)	
Conditioning					1.0
RIC	51 (100)	17 (100)	17 (100)	17 (100)	
TBI-based	19 (37)	10 (59)	5 (29)	4 (24)	0.07
Drug-based	32 (63)	7 (41)	12 (71)	13 (76)	
GvHD prophylaxis					< 0.01
ATG-CsA-MMF	33 (65)	17 (100)	16 (94)	0 (0)	
ATG-Tac-MMF	1 (2)	0 (0)	1 (6)	0 (0)	
CY-Tac-MMF	17 (33)	0 (0)	0 (0)	17 (100)	
Year of transplant					0.92
Median	2013	2013	2012	2013	

Values in parentheses represent percentages if not indicated otherwise.

*ATG*, anti-thymocyte globulin; *BM*, bone marrow; *BW*, body weight; *CMV*, cytomegalovirus; *CsA*, ciclosporin A; *CY*, cyclophosphamide; *Haplo*, haploidentical donor; *HLA*, human leukocyte antigen; *kg*, kilogram; *MMF*, mycophenolate mofetil; *MRD*, matched related donor; *MUD*, matched unrelated donor; *NC*, nucleated cells; *PBSC*, peripheral blood stem cells; *RIC*, reduced intensity conditioning; *Tac*, tacrolimus; *TBI*, total body irradiation.

### Engraftment and DLI

No primary graft rejection occurred (**Supplementary Table 1**). Primary neutrophil engraftment was achieved in all but three patients – two with autologous recovery and persistence of blasts (MRD:1, MUD:1) and one early death in aplasia post Haplo-HSCT. Platelet recovery was significantly delayed in the Haplo-HSCT (Haplo: 40, MRD: 13, MUD: 19 days; p<0.01). Regarding DLI application, four patients were able to receive DLIs (MRD: 2; MUD: 2). All other patients were not eligible for prophylactic DLI because of either early death/early relapse (n = 9), history of or ongoing GvHD including overall grade II aGvHD requiring systemic steroid treatment (n = 34), or recurrent infections (n = 4).

### Immune reconstitution

Pre-transplant lymphocyte subsets were comparable across transplantation platforms (**Table 3**). By day +30, absolute lymphocyte counts (ALC) were significantly lower in Haplo recipients receiving PTCY compared with ATG-treated MRD/MUD patients (p<0.01), however normalized by one year post-HSCT. Differences in ATG dosing between the MRD and MUD groups were not associated with differences in early ALC recovery.

**Table 3:** Immune reconstitution.

	MRD	MUD	Haplo	P-value Global	P-value MRD vs MUD/MRD vs Haplo/ MUD vs Haplo
Pre-Transplantion					
CD45+	900	1090	855	0.63	0.46/0.40/0.75
CD3+	810	740	690	0.52	0.37/0.31/0.88
CD3+/CD4+	380	400	285	0.45	0.94/0.33/0.25
CD3+/CD8+	360	250	260	0.27	0.12/0.29/0.72
CD16+/CD56+	80	130	130	0.45	1.00/0.22/0.38
CD19+	70	40	50	0.81	0.50/0.73/0.90
Day +30					
CD45+	480	520	195	0.02	1.0/<0.01/0.01
CD3+	410	245	115	0.11	0.76/0.04/0.15
CD3+/CD4+	55	25	50	0.40	0.20/0.44/0.57
CD3+/CD8+	200	160	45	0.20	0.44/0.08/0.37
CD16+/CD56+	150	160	30	0.08	0.82/0.09/0.04
CD19+	10	10	7	0.45	0.80/0.58/0.22
Day +100					
CD45+	1168	675	510	0.09	0.20/0.02/0.50
CD3+	750	260	130	0.01	0.07/<0.01/0.17
CD3+/CD4+	135	34	70	0.36	0.19/0.25/0.87
CD3+/CD8+	490	181	85	0.07	0.12/0.02/0.45
CD16+/CD56+	275	185	250	0.85	0.56/0.81/0.82
CD19+	50	17	45	0.65	0.54/0.96/0.37
Day +180					
CD45+	1650	990	1010	0.13	0.09/0.08/0.84
CD3+	1488	420	450	0.06	0.09/0.03/0.45
CD3+/CD4+	180	190	120	0.41	0.43/0.26/0.46
CD3+/CD8+	855	240	320	0.21	0.17/0.10/0.82
CD16+/CD56+	230	200	340	0.50	0.74/0.52/0.26
CD19+	250	70	160	0.43	0.31/0.31/0.53
Day +360					
CD45+	1500	1710	1200	0.79	0.44/0.75/0.95
CD3+	830	750	620	0.92	0.80/0.95/0.70
CD3+/CD4+	180	150	170	0.69	0.55/0.65/0.46
CD3+/CD8+	590	300	470	0.71	0.46/0.75/0.60
CD16+/CD56+	200	250	220	0.35	0.23/0.27/0.66
CD19+	165	160	195	0.38	0.66/0.17/0.50

*Haplo*, haploidentical donor; *MRD*, matched related donor; *MUD*, matched unrelated donor.

Focusing on day +30 on total CD3+ T cells, the counts were markedly reduced after PTCY (median 115/µl) compared to MUD (245/µl) and MRD (410/µl; **Table 3**). The largest divergence between platforms occurred at day +100, with MRD recipients showing robust T cell recovery (≈750/µl), while MUD and Haplo groups remained substantially lymphocytopenic (260/µl and 130/µl; p = 0.01). Full T cell reconstitution was achieved by day +100 in MRD-HSCT, but not until day +360 in both MUD- and Haplo-HSCT.

Subset analyses revealed that CD4+ T cells failed to reach normal levels in any cohort within the first post-transplant year (MRD: 180/µl, MUD: 150/µl, Haplo: 170/µl), while CD8+ T cell counts did not differ significantly across all platforms by day +180. Early NK cell recovery tended to be lower in PTCY-treated patients compared with ATG-treated cohorts (day +30: Haplo: 30/µl vs MRD/MUD: 150/160/µl; p = 0.08). In contrast, B cell reconstitution was delayed across all three groups but approached to normal levels by approximately six months (**Table 3**). Collectively, these data highlight a delay in both innate and adaptive IR following PTCY-based GvHD prophylaxis.

### Infections and toxicities

Rates of CMV-reactivation were similar among patients at risk (MRD n = 8/13, MUD n = 4/7, Haplo n = 9/14; p = 0.95), but the time to reactivation was shortest in MUD recipients (26 days (20–46)) and longest in the Haplo cohort (48 days (10–59); MRD 41 days (27–79)). No CMV prophylaxis was used. One EBV reactivation was observed. One disseminated adenovirus disease (MRD group) was seen and responded to reduction of immunosuppression and cidofovir. Proven and possible invasive fungal infections (IFIs) occurred at moderate rates (*aspergillus fumigatus* n = 7; *aspergillus terreus* n = 1; *geotrichum capitatum* n = 1) and did not cluster by donor type. Toxicities were predominantly grades I-II and did not differ substantially across the three platforms (**Supplementary Table 2**).

### Association between GvHD and IR

**Figure 1:**
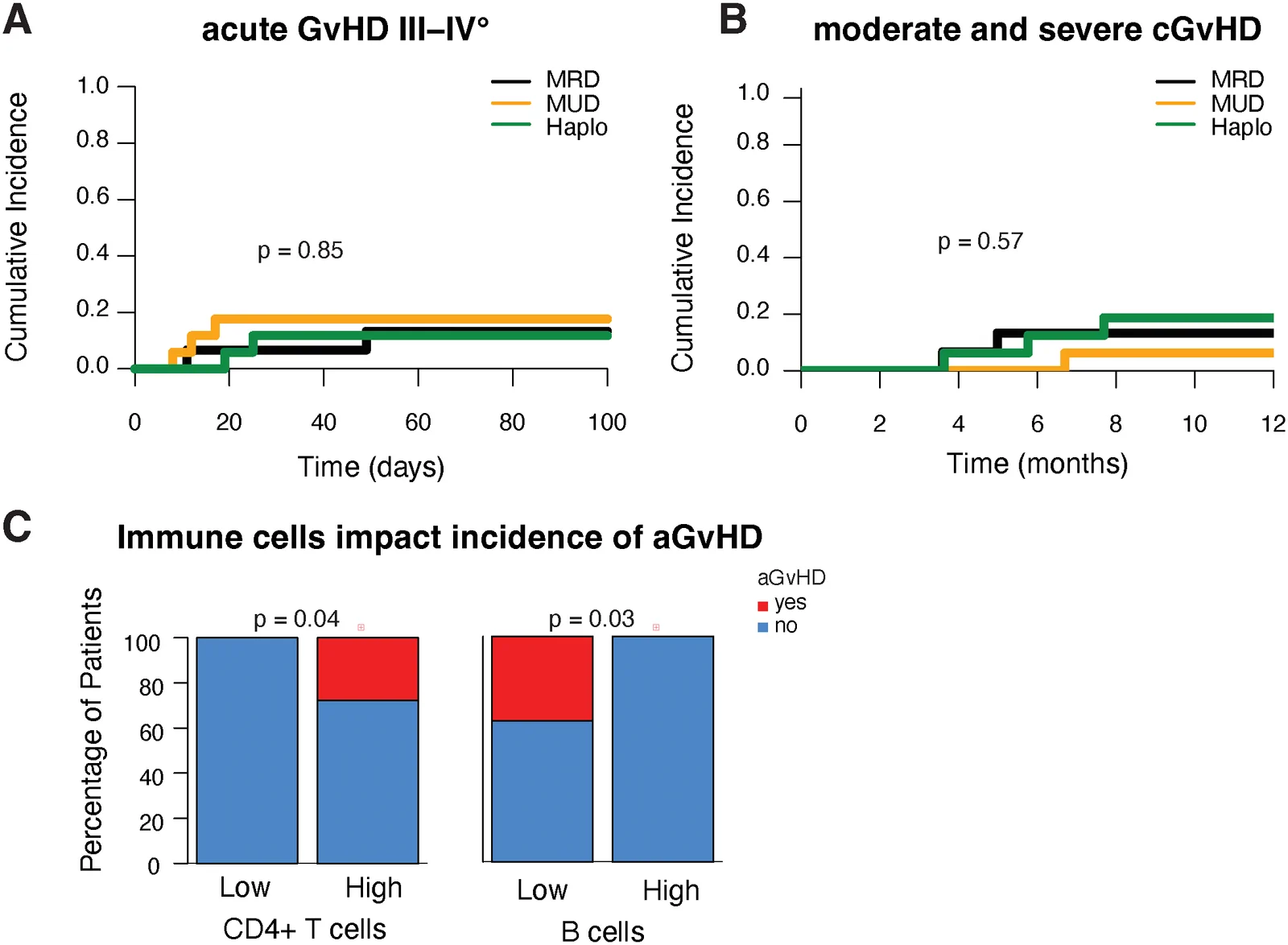
Cumulative incidences of acute GvHD **(A)** and chronic GvHD **(B)** by transplantation platform. **(C)** Stacked bar charts show that aGvHD III-IV° occurs more frequently in patients exhibiting high CD4+ T cell and low B cell counts.

CI of aGvHD (III-IV°) did not differ among the three groups (MRD: 13%, MUD: 19% and Haplo 13%; p = 0.85, **Figure 1A**; **Supplementary Table 3**). The median onset of aGvHD and of cGvHD was earlier in MRD- and MUD-HSCT compared to Haplo-HSCT (aGvHD: 17 vs. 16.5 vs. 27.5 days; cGvHD: 136 vs. 100 vs. 216 days). The 1-y CI of moderate and severe cGvHD was 13%, 6% and 19%, respectively, and did not show a significant difference between the groups (**Figure 1B**, p = 0.57).

Given the interplay between GvHD and IR, we subsequently analyzed potential correlations between specific immune cell subsets and the CI of acute and chronic GvHD. Patients with a higher CD4+ T cell count at day +30 (> 35/µl vs <35/µl) were significantly more likely to develop aGvHD III-IV° (p = 0.04, **Figure 1C**). Low B cell reconstitution by day +100 (< 50/µl vs. > 50/µl) was observed in patients who more frequently developed aGvHD III-IV° (p = 0.03, **Figure 1C**). Moreover, patients with lower B cell counts six months after allo-HSCT tended to have higher incidences of moderate and severe cGvHD (p = 0.1, data not shown). Interestingly, CD8+ T cell recovery had no measurable effect on GvHD risk.

### Association between NRM and relapse with IR

**Figure 2:**
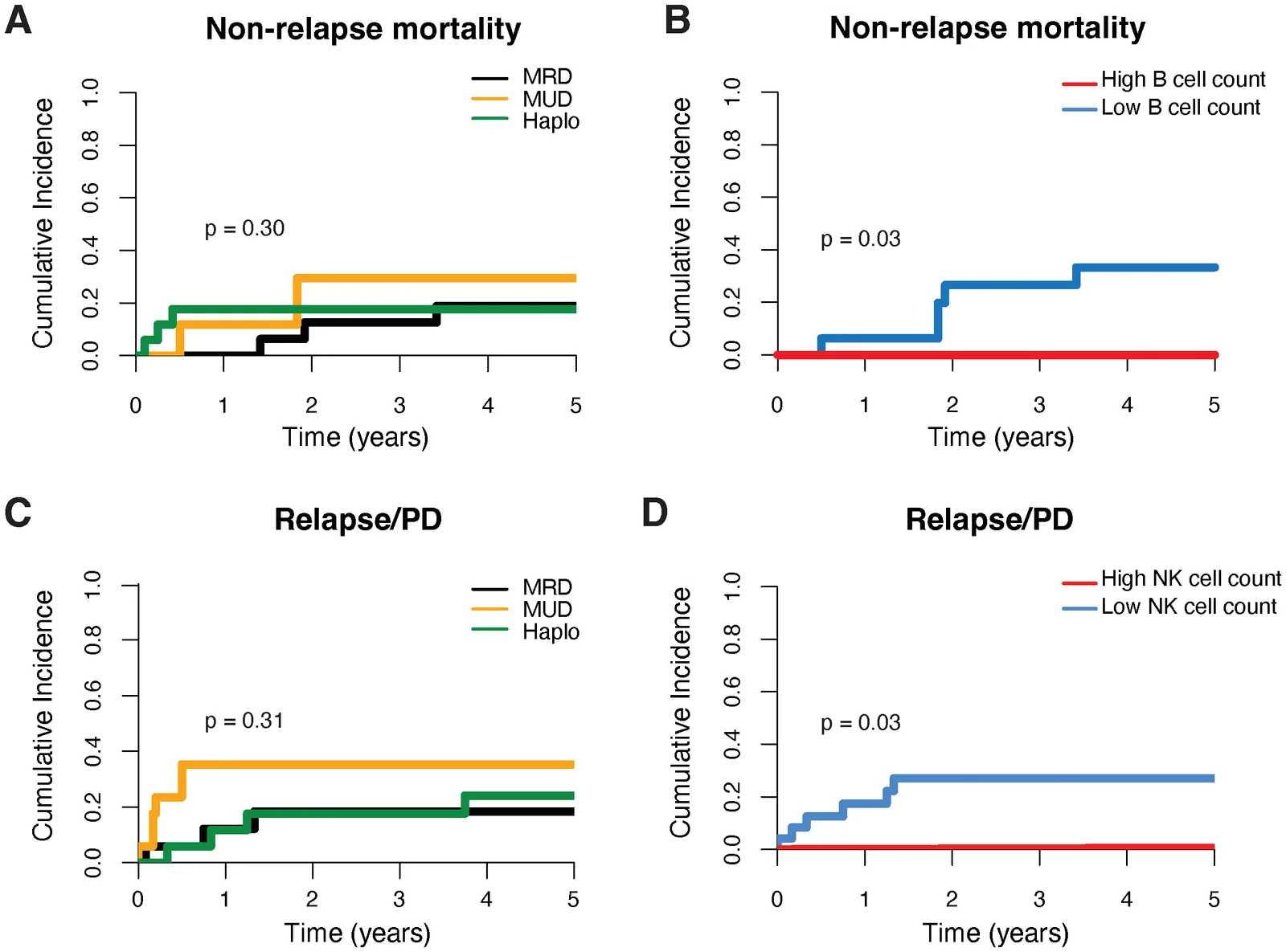
Cumulative incidence of non-relapse mortality by transplantation platform **(A)** or by B cell count 1 year after allo-HSCT **(B)**. Cumulative incidence of relapse or progression of disease (PD) by transplantation platform **(C)** or by NK cell count 1 year after allo-HSCT **(D)**.

CI of NRM at day +100 was low across all groups (CI NRM: 0-6%; p = 0.66, **Supplementary Table 4**). At 1-year, CI of NRM remained 0% in MRD recipients and increased to 12% in MUD and 18% in Haplo (p = 0.38). By 5-years CI of NRM converged to 19%, 29% and 18%, respectively (**Figure 2A**). Whereas in the Haplo group, NRM events occurred early and were infection-related (*pseudomonas putida* sepsis, CMV and Enterobacter cloacae), NRM in MUD recipients occurred later and were mainly GvHD-associated. Notably, delayed B cell recovery after one year was associated significantly with increased NRM (p = 0.03; **Figure 2B**), predominantly in HLA-matched transplants.

CI of relapse at 1-and 5-years did not differ significantly between transplantation platforms (MRD: 12%/18%; MUD: 35%/35%; Haplo: 12%/24%; p = 0.31; **Figure 2C**; **Supplementary Table 4**). While relapse was not associated with early T or B cell abundance, higher NK cell levels (<0.25 vs. >0.75 quantile) at 1 year after transplantation were linked to lower relapse risk (p = 0.03, **Figure 2D**), underscoring the contribution of late innate reconstitution to disease control.

### Survival

**Figure 3:**
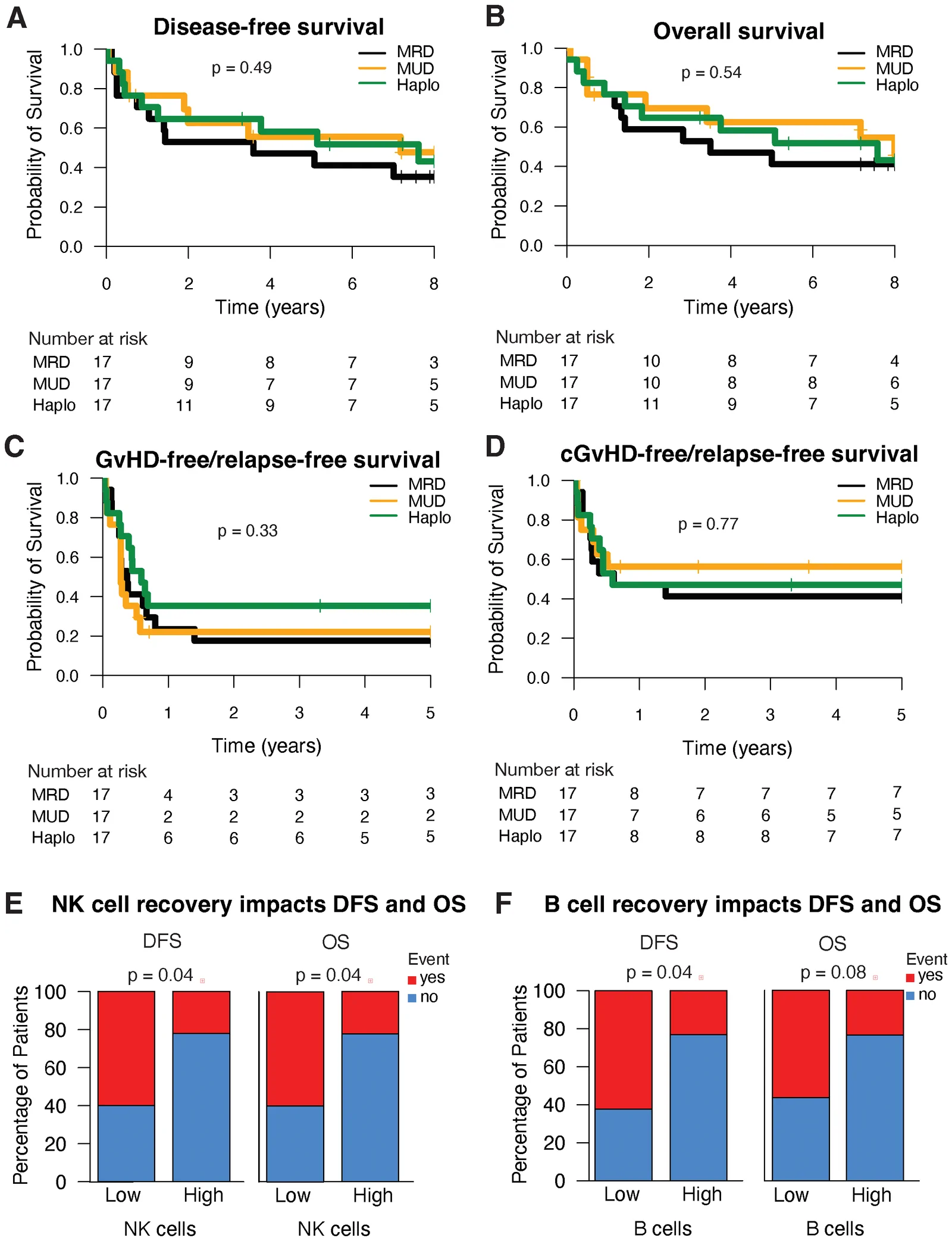
Kaplan–Meier estimates of disease-free survival **(A)**, overall survival **(B)**, GvHD-free and relapse-free survival (GRFS) **(C)** and chronic GvHD-free and relapse-free survival (CRFS) **(D)** by transplantation platform. Stacked bar plots show that patients with lower NK cell **(E)** and B cell counts **(F)** are enriched in DFS and OS events.

With a median follow-up of 8.4, 10.8 and 9.9 years for MRD, MUD and Haplo recipients, respectively, no significant difference was observed in 1-, 3- and 5-year DFS (MRD: 71% (Confidence interval (95% CI) 52-96%)/53% (95% CI 34-83%)/41% (23-73%); MUD 77% (95% CI 59-100%)/63% (95% CI 43-92%)/56% (36-87%); Haplo 71% (95% CI 52-96%)/65% (95% CI 46-92%)/58% (95% CI 39-87%) and 1-, 3- and 5-year OS (MRD: 77% (95% CI 59-100%)/53% (95% CI 34-83%)/41% (23-73%); MUD 77% (95% CI 59-100%)/70% (95% CI 50-96%)/63% (43-92%); Haplo 77% (95% CI 59-100%)/65% (95% CI 46-92%)/58% (95% CI 39-87%); **Figures 3A, B**; **Supplementary Table 4**). Additionally, no significant difference was detected in 1-, 3- and 5-year GRFS and CRFS (**Figures 3C, D**; **Supplementary Table 4**). At the 8-year long-term follow-up, we also found no significant differences in OS and DFS. In the 5-year GRFS analysis, relapse, NRM, aGvHD and cGvHD were similarly distributed between Haplo and MUD allo-HSCT, with cGvHD accounting for most events in both groups (**Supplementary Figure 1A**). First CRFS events are given in **Supplementary Figure 1B** and show a similar distribution across transplantation platforms.

Analysis of the impact of IR on post-transplant outcomes demonstrated a significant survival advantage among patients with higher NK cell counts at 1 year, with improvements observed in both DFS and OS (p = 0.04/0.04, **Figure 3E**). Similarly, patients with higher B cell counts 1 year after allo-HSCT showed superior DFS (p = 0.04) and a trend toward improved OS (p = 0.08, **Figure 3F**).

## Discussion

By performing a matched-pair analysis using age, disease status and activity, and co-morbidity scores as matching criteria, our study provides a comprehensive and detailed assessment of different sequential conditioning HSCT platforms in older patients with advanced myeloid disease. We specifically focused on IR after sequential regimens and its impact on clinical outcomes.

In line with previous data in younger patient cohorts (1, 3, 26), our results demonstrate that sequential conditioning allo-HSCT is safe and feasible in heavily pretreated elderly patients with multiple co-morbidities and leads to durable disease control (3-year DFS: 53% MRD-HSCT, 63% MUD-HSCT, 65% Haplo-HSCT). Compared to outcomes reported in elderly patients undergoing non-sequential matched allo-HSCT (27), our sequential approach improves both survival and long-term disease control (8-year OS: MRD 41%, MUD 46%, Haplo 43%; 8-year DFS: MRD 35%, MUD 48%, Haplo 43%).

Previous studies have shown that lymphocyte subset recovery at defined time points after transplant is a strong predictor of survival and NRM, making effective IR an important goal in allogeneic transplantation (28–32). The concern that extended cytoreductive therapy prior to HSCT may delay overall IR was not supported by our findings. In our study, the sequential regimen resulted in IR patterns comparable to those seen with non-sequential approaches (33). Moreover, in line with McCurdy and Luznik (34), we did not observe a general impaired immune cell recovery after Haplo-HSCT. The immune recovery patterns were largely comparable across our MRD-, MUD- and Haplo-HSCT. However, early post-HSCT recovery was delayed in Haplo-HSCT, likely related to the use of PTCY and prolonged immunosuppression (35, 36). Notably, this delay diminished over time. Although the NRM did not differ across the three platforms and was comparable to rates in younger cohorts (1, 3, 26), higher rates of early infections were observed in the Haplo-HSCT. Chang et al. also reported that low ALC at day +100 in the Haplo setting using the GIAC protocol was linked to earlier and higher bacterial infections (37), highlighting the importance of timely IR in the Haplo setting. Achieving this requires consideration of several factors such as the use and dose of PTCY, the impact of prolonged immunosuppression and the choice of BM as a graft source. Of note, the use of DLI was limited in our cohort, likely reflecting a cautious clinical approach in the context of GvHD, where the risk of exacerbation often precludes DLI administration. Whether a more refined and risk-adapted use of DLI in the setting of sequential therapy could further improve disease and viral infection control without increasing GvHD remains to be determined. Large registry studies and CIBMTR analyses are ongoing to better define the risk of early bacterial, fungal, and viral infections under different prophylaxis regimens and graft-source strategies. One promising approach is PTCY dose reduction, aiming to limit the initial lymphocyte depletion and accelerate immune recovery. Recent dose de-escalation studies have shown feasibility, maintained GvHD control, and faster lymphocyte reconstitution compared with standard PTCY (38–40). Of note, our cohort was transplanted before letermovir became available, and routine CMV prophylaxis would likely mitigate the early infection-related events seen here. Together with the ongoing evolution of PTCY protocols and graft source strategies, this may narrow the early IR differences observed between platforms.

CIs of acute and chronic GvHD were not statistically different between the platforms, although such differences have been described previously (41). Analyzing the complex bidirectional interplay between immune recovery and GvHD (42), our data support the observation that early higher numbers of CD4+ T cells might aggravate aGvHD (43). We also observed that patients with aGvHD exhibited delayed B cell recovery, suggesting that both aGvHD and immunosuppressive treatments like steroids may hinder B cell regeneration (44, 45). However, published data on the association between not-antibody-mediated aGvHD and B cells are inconsistent, and the causal relationship remains unclear (46). In contrast, stronger and more consistent evidence links low B cell recovery to cGvHD. Consistent with prior research, our study shows that patients with cGvHD tend to have impaired or delayed B cell reconstitution compared to those without cGvHD (47). These findings support the idea that monitoring B cell subsets after HSCT could serve as a useful biomarker for GvHD risk.

In contrast to major concerns regarding Haplo-HSCT, relapse incidence seems not to be influenced by delayed IR or prolonged use of immunosuppressants. Interestingly, we observed that after initial depletion by PTCY in the Haplo-HSCT regimen protocols, NK cells seem to expand robustly and ultimately reached comparable levels in haploidentical recipients compare to HLA-matched patients. Moreover, we observed that higher NK cell levels were associated with a significantly lower CI of relapse and superior DFS and OS, in line with previous reports (31) suggesting that NK cells may serve as a functionally relevant component of graft-versus-leukemia activity. Strategies aiming to augment NK-cell recovery and function, such as cytokine support or adoptive NK-cell transfer, may further improve disease control without increasing GvHD incidences.

We are fully aware of the shortcomings of the study including the retrospective design and the small size. With 17 patients per donor group, statistical power was limited, comparative analyses are exploratory, and negative findings may reflect limited power rather than true equivalence. Additionally, a more suitable comparison would involve using the same graft source and GvHD prophylaxis across all donor types. This was not feasible at our center, as transplantation strategies adhered to standard practices for MRD and MUD. Nevertheless, this study provides a detailed comparison of clinical outcome, GvHD, infection, treatment-related toxicities, and their association with IR, without the confounding influence of institutional expertise or local practice variations.

In conclusion, sequential therapy proved to be a safe and feasible option for elderly patients with high-risk and non-remission disease across all three transplant platforms. By examining the impact of IR on clinical outcomes, our study offers a comprehensive overview of the interplay between IR and major outcome parameters. Prospective studies in larger cohorts are needed to substantiate these associations. A deeper understanding of these immunologic dynamics may not only improve strategies to reduce both GvHD and relapse but may also give important insights for the development of novel immune-based therapeutic approaches for patients who experience relapse after allo-HSCT.

## Data Availability

The original contributions presented in the study are included in the article/**Supplementary Material**. Further inquiries can be directed to the corresponding author.
