# Supplementary material for: Sequential conditioning in hematopoietic stem cell transplantation in elderly high-risk myeloid blood cancer patients: long-term survival, disease control, and immune recovery across donor types in a matched-pair analysis
Source: Front Immunol. 2026 Aug 26;17:1803250. doi: 10.3389/fimmu.2026.1803250 (PMC13553495; doi:10.3389/fimmu.2026.1803250)
Supplement: Supplementary file 1 [file DataSheet1.docx]

Supplementary Material

Table of Contents

[1	Supplementary Tables	2](#_Toc220856615)

[2	Supplementary Figures	6](#_Toc220856616)

# 1 Supplementary Tables

**Supplementary Table 1.** **Graft rejection and engraftment**

|  | **MRD** | **MUD** | **Haplo** | **P-value** |
| --- | --- | --- | --- | --- |
| **Evaluable patients** | 17 | 17 | 16 |  |
| **Graft rejection** | 0 | 0 | 0 | 1.0 |
| ANC > 500/µl | 16 | 16 | 16 |  |
| Median days (range) | 16 (10-30) | 16 (11-43) | 18 (13-89) | 0.26 |
| PLT > 20,000/µl * | 16 | 15 | 15 |  |
| Median days (range) | 13 (10-34) | 19 (13-37) | 40 (11-103) | <0.01 |

*Platelet recovery, defined as achieving a count of more than 20,000 cells/µl for three consecutive days without transfusion

*ANC*, absolute neutrophil count; *Haplo,* haploidentical donor; *MRD,* matched related donor*; MUD,* matched unrelated donor*; PLT*, platelets.

**Supplementary Table 2.** **Treatment-related toxicity**

|  | **MRD** | **MUD** | **Haplo** | **P-value** |
| --- | --- | --- | --- | --- |
| **Mucositis** | 7 (41) | 8 (47) | 10 (59) | 0.56 |
| Grade I-II | 4 (24) | 3 (18) | 6 (35) |  |
| Grade III-IV | 3 (18) | 5 (29) | 4 (24) |  |
| **Diarrhea** | 14 (82) | 16 (94) | 17 (100) | 0.15 |
| Grade I-II | 9 (53) | 8 (47) | 6 (35) |  |
| Grade III-IV | 5 (29) | 8 (47) | 11 (65) |  |
| **Arrhythmia** | 2 (12) | 4 (24) | 4 (24) | 0.12 |
| **Acute renal failure** | 7 (41) | 6 (35) | 4 (24) | 0.54 |
| Requiring hemodialysis | 2 (12) | 1 (6) | 2 (12) |  |
| **Hemorrhagic cystitis** | 0 | 2 (12) | 2 (12) | 0.34 |
| **Elevated bilirubin** | 10 (59) | 12 (71) | 2 (12) | < 0.01 |
| Grade I-II | 8 (47) | 9 (53) | 2 (12) |  |
| Grade III-IV | 2 (12) | 3 (18) | 0 |  |
| **Elevated serum transaminases** | 6 (35) | 5 (29) | 6 (35) | 0.91 |
| Grade I-II | 5 (29) | 5 (29) | 4 (24) |  |
| Grade III-IV | 1 (6) | 0 | 2 (12) |  |
| **VOD** | 0 | 0 | 0 |  |
| **Rash** | 2 (12) | 3 (18) | 8 (47) | 0.04 |
| **Hand-foot syndrome** | 0 | 0 | 2 (12) | 0.11 |
| **Confusion** | 3 (18) | 2 (12) | 4 (24) | 0.45 |
| **Fever** | 16 (94) | 16 (94) | 17 (100) | 0.59 |
| **TAM** | 2 (12) | 0 | 2 (12) | 0.34 |

Values in parentheses represent percentages if not indicated otherwise.

*Haplo*, haploidentical donor; *MRD*, matched related donor; *MUD*, matched unrelated donor; *TAM,* transplantation-associated microangiopathy; *VOD,* veno-occlusive disease.
**Supplementary Table 3. Graft-versus-host disease**

|  | **MRD** | **MUD** | **Haplo** | **P-value** |
| --- | --- | --- | --- | --- |
| **Acute GvHD*** | 12 (75) | 12 (75) | 14 (88) |  |
| I-II | 10 (63) | 9 (56) | 12 (75) | 0.52 |
| III-IV | 2 (13) | 3 (19) | 2 (13) | 0.85 |
| **Chronic GvHD **** | 9 (60) | 9 (56) | 7 (44) |  |
| Mild | 7 (47) | 8 (50) | 4 (25) | 0.30 |
| Moderate | 2 (13) | 1 (6) | 2 (13) | 0.57 |
| Severe | 0 (0) | 0 (0) | 1 (6) |  |

Values in parentheses represent percentages if not indicated otherwise. *Evaluable patients: MRD: 16, MUD: 16, Haplo: 16. **Evaluable patients: MRD: 15, MUD: 16, Haplo: 16

*GvHD*, Graft-versus-host disease; *Haplo,* haploidentical donor; *MRD,* matched related donor*; MUD,* matched unrelated donor.
**Supplementary Table 4.** **Outcomes, non-relapse mortality and relapse after allo-HSCT**

|  | **MRD** | **MUD** | **Haplo** | **P-value** |
| --- | --- | --- | --- | --- |
| **NRM % (95% CI)** |  |  |  |  |
| CI day +100 | 0 | 0 | 6 (3-9) | 0.66 |
| CI 1-year | 0 | 12 (8-16) | 18 (13-23) | 0.38 |
| CI 5-years | 19 (13-25) | 29 (25-34) | 18 (13-23) | 0.30 |
| **Relapse % (95% CI)** |  |  |  |  |
| CI 1-year | 12 (7-17) | 35 (30-40) | 12 (7-17) | 0.31 |
| CI 3-years | 18 (13-23) | 35 (30-40) | 18 (13-23) | 0.33 |
| CI 5-years | 18 (13-23) | 35 (30-40) | 24 (18-30) | 0.31 |
| **Overall survival % (95% CI)** | |  |  |  |
| 1-year | 77 (59-100) | 77 (59-100) | 77 (59-100) | 0.57 |
| 3-years | 53 (34-83) | 70 (50-96) | 65 (46-92) | 0.51 |
| 5-years | 41 (23-73) | 63 (43-92) | 58 (39-88) | 0.55 |
| 8-years | 41 (23-73) | 46 (25-82) | 43 (24-78) | 0.54 |
| **Disease-free survival % (95% CI)** | |  |  |  |
| 1-year | 71 (52-96) | 77 (59-100) | 71 (52-96) | 0.53 |
| 3-years | 53 (34-83) | 63 (43-92) | 65 (46-92) | 0.48 |
| 5-years | 41 (23-73) | 56 (36-87) | 58 (39-88) | 0.52 |
| 8-years | 35 (19-67) | 48 (28-82) | 43 (24-78) | 0.49 |
| **GRFS % (95% CI)** |  |  |  |  |
| 1-year | 24 (10-55) | 35 (19-67) | 35 (19-67) | 0.33 |
| 3-years | 18 (6-49) | 28 (13-62) | 35 (19-67) | 0.33 |
| 5-years | 18 (6-49) | 21 (8-55) | 35 (19-67) | 0.33 |
| **CRFS % (95% CI)** |  |  |  |  |
| 1-year | 47 (28-78) | 56 (37-87) | 47 (28-78) | 0.77 |
| 3-years | 41 (23-73) | 56 (37-87) | 47 (28-78) | 0.77 |
| 5-years | 41 (23-73) | 56 (37-87) | 47 (28-78) | 0.77 |

*CI,* cumulative incidence; *CRFS*, chronic graft-versus-host disease and relapse-free survival; *GRFS*, graft-versus-host disease and relapse-free survival; *Haplo,* haploidentical donor*; MRD,* matched related donor*; MUD,* matched unrelated donor*; NRM*, non-relapse mortality.

# 2 Supplementary Figures

**Supplementary Figure 1. GRFS and CRFS events.**

Relative distribution of GvHD-free/relapse-free survival (GRFS) events (**A**) and cGvHD-free/relapse-free survival (CRFS) events (**B**) including relapse, non-relapse mortality (NRM), acute graft-versus-host disease (aGvHD), and chronic GvHD (cGvHD) in matched HLA-related donor (MRD), matched HLA-unrelated donor (MUD), and HLA-haploidentical donor (Haplo) transplantation.
